# Revisiting anthelmintic resistance in sheep flocks from São Paulo State, Brazil

**DOI:** 10.1016/j.ijpddr.2024.100527

**Published:** 2024-02-28

**Authors:** Cesar C. Bassetto, Ana Cláudia A. Albuquerque, José Gabriel G. Lins, Naiara M. Marinho-Silva, Marianna L.E. Chocobar, Hornblenda J.S. Bello, Mateus O. Mena, Simone C.M. Niciura, Alessandro F.T. Amarante, Ana Carolina S. Chagas

**Affiliations:** a Southeast Livestock Unit, Brazilian Agricultural Research Corporation (EMBRAPA), São Carlos, SP, Brazil; b School of Agricultural and Veterinary Sciences, São Paulo State University (UNESP), Jaboticabal, SP, Brazil; c School of Veterinary Medicine and Animal Science, UNESP, Botucatu, SP, Brazil; d Department of Biodiversity and Biostatistics, Institute of Biosciences, UNESP, Botucatu, SP, Brazil

**Keywords:** Animal production, Epidemiology, Faecal egg count reduction test, Gastrointestinal nematodes, *Haemonchus contortus*, Multiple resistance

## Abstract

*Haemonchus contortus* and *Trichostrongylus colubriformis* are the most important gastrointestinal nematodes causing serious losses in sheep production of tropical and subtropical regions. Prophylaxis of gastrointestinal nematode infections is based on anthelmintics use, but their frequent administration selects multiple-resistant parasites. To evaluate how the situation has changed over the last decades, the anthelmintic resistance status of gastrointestinal nematodes in sheep flocks was assessed in the current study and compared to previous surveys. In each one of the 15 flocks evaluated, animals (n ≥ 7) were allocated into at least five groups and treated as follows: 1) untreated control; 2) albendazole; 3) levamisole; 4) ivermectin; and 5) monepantel. If more animals were available, two additional groups were included: 6) closantel, and 7) moxidectin. The faecal egg count reduction test (FECRT) was carried out to evaluate the pre- and post-treatment using the SHINY tool. *Haemonchus* spp. was the most prevalent nematode from faecal cultures. The mean efficacy of albendazole was 40%. Only in two farms, levamisole presented a relatively high percentage of reduction in the FECRT about 90%, while ivermectin and moxidectin presented the worst mean efficacy of 34% and 21% among all farms, respectively. Like other anthelmintics, closantel demonstrated low efficacy (63%) across all farms evaluated. Monepantel presented an overall mean efficacy of 79%, but it was the only anthelmintic that presented efficacy ≥95%, in five farms. The results revealed that gastrointestinal nematodes with multiple anthelmintic resistance were prevalent in all 15 sheep herds. The research suggests that nematodes are becoming more and more resistant to various anthelmintic compounds, which has made the problem worse. This circumstance highlights the necessity to put into practice sustainable and long-lasting methods to prevent gastrointestinal nematode infections in sheep husbandry.

## Highlights

• Multiple anthelmintic resistance is established in all sheep farms evaluated.
• Anthelmintic resistance status has worsened over the last three decades.
• Monepantel was 100% effective against *Trichostrongylus* spp.
• *Haemonchus* spp. and *Trichostrongylus* spp. are the most resistant genera.
• *Oesophagostomum* spp. is the most susceptible genus.

## Graphical abstract

**Figure:**
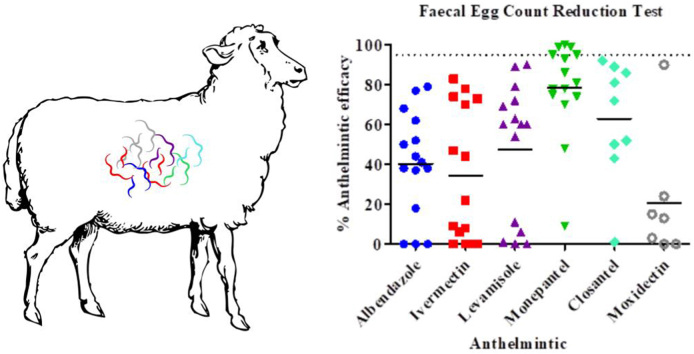

## 1 Introduction

The main health problems in sheep flocks are caused by gastrointestinal nematode infections, which comprise several parasite species. In tropical and subtropical regions, *Haemonchus contortus* is highly prevalent and infective, causing anaemia, dyspepsia and high morbidity and also mortality of sheep due to its hematophagous habits (Amarante et al., 2004; Rocha et al., 2004; Wilmsen et al., 2014; Besier et al., 2016). Moreover, it presents high biotic potential, as the parasite stage can survive for nearly 1.5 years in the host (Santos et al., 2014) and the free-living stages survive for a long period in the field (Almeida et al., 2020). The second most prevalent gastrointestinal nematode of sheep in Brazil is *Trichostrongylus colubriformis* (Wilmsen et al., 2014; Almeida et al., 2018), which causes weight losses, morbidity, and a reduction in food intake (Kimambo et al., 1988; Carvalho et al., 2021).

The prophylaxis of ovine nematode infections is mostly based on the use of anthelmintics. Adversely, because of their frequent use, anthelmintic resistance reports have increased around the world (reviewed by Gilleard et al., 2021). In Brazil, the status of anthelmintic resistance is also alarming, as demonstrated in the literature by multiple resistance to all commercially available compounds (Veríssimo et al., 2012; Salgado and Santos, 2016; Albuquerque et al., 2017; Oliveira et al., 2017; Silva et al., 2018; Nagata et al., 2019; reviewed by Macedo et al., 2023).

In Sao Paulo State the more extensive trials to evaluate resistance to anthelmintics were carried out by Amarante et al. (1992) and Veríssimo et al. (2012). These studies reported high levels of resistance to benzimidazoles in all farms, while ivermectin presented efficacy only in 22% of the farms evaluated in the first study, and none in the second study. Levamisole showed high efficacy in 22% and 46% of the farms, respectively. Veríssimo et al. (2012) also reported low levels of efficacy by closantel and moxidectin. Recently, Gainza et al. (2021) reported the situation in five farms, when they compared an *in vivo* and an *in vitro* (RESISTA-Test©) methods aiming to validate the latter for the diagnosis of anthelmintic resistance. Their results confirmed the high degree of resistance to benzimidazoles, ivermectin and levamisole, except monepantel that presented high efficacies.

After decades of new research and the introduction of targeted selective treatment (TST) for example FAMACHA method (van Wyk and Bath, 2002), it was expected that the efficacy of anthelmintics would be preserved, and that the situation would not deteriorate. With the adoption of TST management, it would be possible to reduce the development of anthelmintic resistance, which would contribute to the sustainability of sheep production (Santos et al., 2022). Evaluating the efficacy of TST with levamisole over 5 years, it was observed an increase in the efficacy of the other anthelmintic classes (Chagas et al., 2016). This research was in concordance that switching to less intensive treatment regimens or anthelmintic combinations may be beneficial in reducing or even reversing anthelmintic resistance in gastrointestinal nematode populations (Waller, 1997; Leathwick et al., 2015).

Determining and monitoring the resistance status of gastrointestinal nematodes in sheep against anthelmintic compounds is crucial to establishing rational measures for prevention and control. As outlined above new techniques have been developed over the years that offer sheep farmers other management options, which can be beneficial reducing the anthelmintic resistance in the flocks. Therefore, in the present study, the anthelmintic resistance status of gastrointestinal nematodes in sheep flocks was evaluated and compared to past surveys to investigate the evolution of the situation over the last three decades.

## 2 Materials and methods

**Fig. 1:**
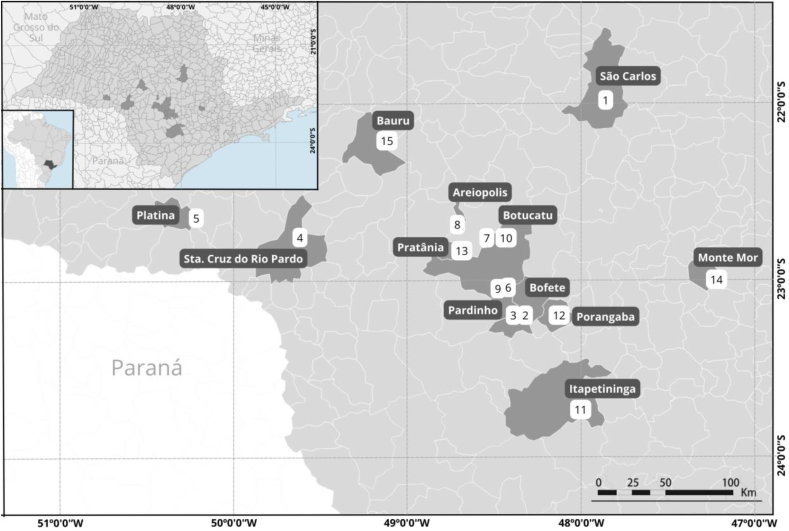
Geographical coordinates of 15 sheep farms evaluated for anthelmintic resistance in São Paulo State, Brazil.

The trial was carried out from March to September 2022 on 15 sheep farms from different localities in the state of São Paulo, Brazil (Fig. 1), including sheep of different breeds, sexes, and ages.

All procedures performed in this study were approved by the Ethics Committee on Animal Use of Embrapa Southwest Livestock (Protocol no. 01/2020).

### 2.1 Animals and experimental design

In each one of the 15 flocks, animals (n ≥ 7) were allocated into at least five groups and treated as follows: 1) untreated control; 2) albendazole (Valbazen® 10 Cobalto oral, 5 mg/kg body weight (BW), Zoetis, Campinas, Brazil); 3) levamisole (Ripercol® L 150 F subcutaneous, 6.2 mg/kg BW, Zoetis, Campinas, Brazil); 4) ivermectin (Ivomec® 1% subcutaneous, 0.2 mg/kg BW, Boehringer Ingelheim, Paulínia, Brazil); 5) monepantel (Zolvix® 2.5% oral, 2.5 mg/kg BW, Elanco, Dundee, UK). In the case of flocks with a larger number of animals available, two additional groups were included: 6) closantel (Diantel oral, 10 mg/kg BW, Hipra Saúde Animal, Porto Alegre, Brazil), and 7) moxidectin (Cydectin® subcutaneous, 0.2 mg/kg BW, Zoetis, Campinas, Brazil). The untreated control group was included to observe any natural decline or increase in the faecal egg counts (FEC) as suggested by Neves et al. (2014).

### 2.2 Faecal egg count reduction test (FECRT)

Faecal samples were collected from the rectum of each animal one day before the drench day (D −1) for FEC and faecal culture. In addition, the animals were weighed individually. To be included in the study, the farms and their respective flocks had to: (1) present a minimum of 7 animals with FEC ≥200 per group; and (2) the animals had not been treated with anthelmintics for at least 30 days before the experiment as recommended by Coles et al. (2006).

The animals were distributed homogeneously, based on weight and FEC, into groups. The sheep were drenched (D0), following each anthelmintic manufacturer's specifications described above. After 14 days (D14), a new faecal sample was collected and used for individual FEC and group faecal culture.

Nematode eggs were enumerated by a modified McMaster method using 2 g of faeces and 28 mL of saturated salt solution, multiplying the number of eggs by 50. Faecal cultures were performed for each group, and the larvae genera were identified (Ueno and Gonçalves, 1998).

### 2.3 Data analysis

The post-treatment faecal egg count reduction (FECR) was estimated using the SHINY tool, a web interface developed by the ‘shiny-eggCounts' project, which provides an intuitive web interface for analysing FEC data (http://shiny.math.uzh.ch/user/furrer/shinyas/shiny-eggCounts/). This tool calculates the efficacy based on FEC pre- and post-treatment within the same group, allowing the analysis of paired samples (Torgerson et al., 2014), as recently indicated by the World Association for the Advancement of Veterinary Parasitology (WAAVP, Kaplan et al., 2023).

Susceptibility was attributed to FECR ≥95% and a lower uncertainty interval ≥90%, while resistance was identified when FECR <95% and a lower uncertainty interval <90%. FEC was described as mean (± standard deviation) and FECR as percentage and 95% uncertainty intervals. Additionally, the percentages of infective larvae (L_3_) of each gastrointestinal nematode genus from faecal cultures were calculated after counting at least 100 L_3_.

Percentage in the larvae reduction test (LRT) after treatment (D14) was calculated using the formula: LRT = 100 × (1 − [L_3_Treated/L_3_Control]), where L_3_Treated is the L_3_ proportion from each treated group and L_3_Control represent the L_3_ proportion from control group. This formula was adapted from Cristel et al. (2017).

## 3 Results

In general, the main sheep breeds raised on the Sao Paulo State farms were Santa Ines, Dorper, Ile de France, Texel, Suffolk, White Dorper, and their crosses. Targeted selective treatment, mostly based on FAMACHA, was applied in 14 out of 15 farms, and the animals were dewormed monthly only in Farm 15. It is important to mention that Farm 15 started to raise crossed Dorper sheep only six months before this trial; there is no information about the origin of the animals.

**Fig. 2:**
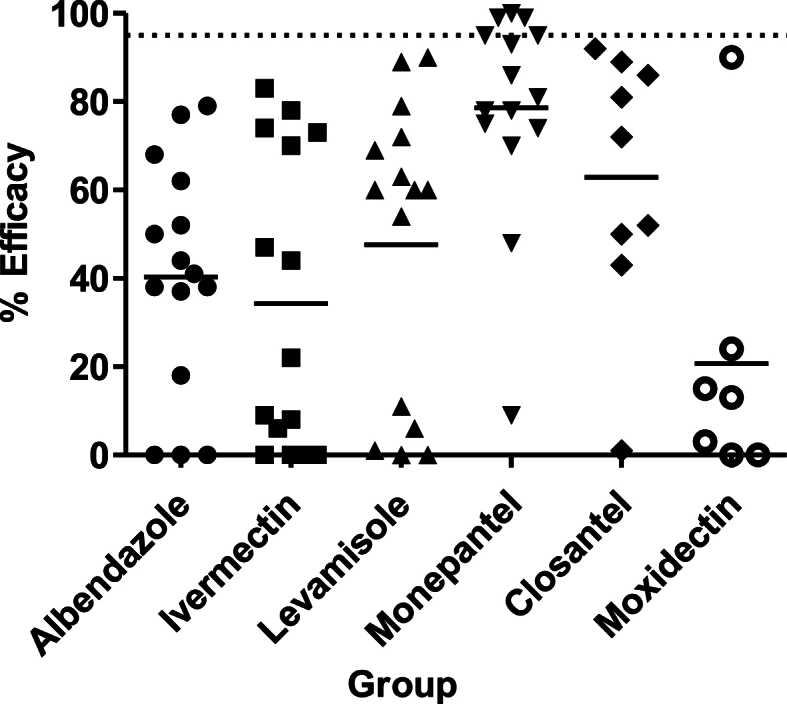
Overall anthelmintic efficacy (%) of albendazole, ivermectin, levamisole, monepantel, closantel and moxidectin based on the faecal egg count reduction test (FECRT) calculated with SHINY tool. Bars represent the mean efficacy, and the dotted line is the threshold of 95% to indicate susceptibility or resistance.

The faecal analyses and FECRT results are described in Table 1 and Fig. 2. Prior to FECRT, considering all farms, the lowest FEC mean was 1026 (±915) and the highest was 6935 (±7768). In general, Farms 12 and 15 showed the highest percentages of efficacy based on FECR, while Farms 3 and 4 notably presented the lowest. The most prevalent (59%–99%) gastrointestinal nematode genus in faecal cultures in control groups was *Haemonchus* spp., except in Farm 10, which presented 88% *Trichostrongylus* spp., and Farm 12, with 53% *Cooperia* spp.

**Table 1:** Mean faecal egg counts ± standard deviation (FEC ± SD) pre- and post-treatment, faecal egg count reduction test (FECRT) with anthelmintic efficacy percentage, lower and upper 95% uncertainty interval (UI) from SHINY, and percentage of parasite genera from faecal cultures 14 days after drench.

Farm	Group	*n*	Mean FEC (±SD)		% FECRT (UI)	Parasite genera (%)			
			Pre-treatment	Post-treatment		H	T	C	O
1	CON	11	1005 ± 889	532 ± 630	–	99	1	0	0
	ALB	9	1167 ± 1129	950 ± 1350	18 (3; 32)	94	6	0	0
	IVM	10	1175 ± 1110	320 ± 461	73 (64; 79)	91	9	0	0
	LEV	11	1023 ± 1055	100 ± 158	90 (85; 94)	99	1	0	0
	MPT	11	1136 ± 1681	1041 ± 1316	9 (0; 21)	100	0	0	0
	CLO	10	1070 ± 1073	85 ± 219	92 (87; 95)	86	14	0	0
	MOX	11	1055 ± 971	105 ± 168	90 (85; 94)	95	5	0	0
2	CON	7	1036 ± 976	2871 ± 3266	–	67	23	5	5
	ALB	7	1014 ± 741	483 ± 759	52 (37; 65)	9	49	42	0
	IVM	7	1036 ± 921	179 ± 182	83 (74; 89)	5	95	0	0
	LEV	7	993 ± 828	407 ± 374	60 (44; 70)	18	82	0	0
	MPT	7	1271 ± 1429	379 ± 651	70 (60; 79)	33	0	6	61
	CLO	7	1671 ± 2514	836 ± 485	50 (38; 60)	0	77	23	0
	MOX		*	*	*	*	*	*	*
3	CON	7	3064 ± 3110	1957 ± 740	–	68	17	4	11
	ALB	7	1850 ± 1273	7521 ± 7959	0 (0; 1)	96	2	1	1
	IVM	7	3729 ± 3398	4650 ± 3845	0 (0; 4)	95	5	0	0
	LEV	7	4707 ± 8576	11,336 ± 24,779	0 (0; 1)	89	10	1	0
	MPT	7	5564 ± 7024	1443 ± 1674	74 (70; 78)	45	0	1	54
	CLO	7	3821 ± 4142	2171 ± 2410	43 (34; 50)	83	15	1	1
	MOX		*	*	*	*	*	*	*
4	CON	8	1938 ± 1886	3131 ± 3031	–	96	4	0	0
	ALB	8	1831 ± 1884	9913 ± 9539	0 (0; 0)	100	0	0	0
	IVM	8	1800 ± 2969	4638 ± 3590	0 (0; 1)	93	7	0	0
	LEV	8	2250 ± 3199	3156 ± 2340	0 (0; 4)	48	52	0	0
	MPT	8	1894 ± 3150	956 ± 1261	48 (39; 58)	100	0	0	0
	CLO		*	*	*	*	*	*	*
	MOX		*	*	*	*	*	*	*
5	CON	7	4471 ± 4895	2843 ± 3339	–	59	18	5	18
	ALB	7	7279 ± 12,832	1507 ± 1864	79 (76; 82)	92	5	3	0
	IVM	7	7321 ± 11,111	8579 ± 16,023	0 (0; 3)	78	21	1	0
	LEV	7	8714 ± 16,610	1829 ± 1988	79 (76; 82)	89	11	0	0
	MPT	7	6571 ± 9391	950 ± 1281	86 (83; 88)	90	0	5	5
	CLO	7	6129 ± 8493	2950 ± 4399	52 (45; 57)	85	9	3	3
	MOX	7	6100 ± 8499	5121 ± 7481	15 (7; 24)	93	1	6	0
6	CON	8	2059 ± 1870	3125 ± 4903	–	91	7	0	2
	ALB	9	3322 ± 5501	2061 ± 2791	38 (30; 46)	97	3	0	0
	IVM	9	3017 ± 2994	2772 ± 2512	9 (0; 17)	95	5	0	0
	LEV	9	2656 ± 3910	2356 ± 1811	11 (1; 21)	87	13	0	0
	MPT	9	2306 ± 2219	0 ± 0	100 (99; 100)	9	0	0	91
	CLO	9	2117 ± 2729	300 ± 412	86 (81; 89)	2	98	0	0
	MOX	9	2356 ± 2354	7294 ± 9892	0 (0; 1)	90	10	0	0
7	CON	7	1436 ± 1212	6971 ± 12,888	–	59	40	1	0
	ALB	7	1307 ± 924	814 ± 742	37 (21; 51)	47	53	0	0
	IVM	7	2057 ± 2823	636 ± 445	70 (61; 76)	46	51	3	0
	LEV	7	2436 ± 3895	2279 ± 1960	6 (0; 18)	65	35	0	0
	MPT	7	1871 ± 2103	343 ± 609	81 (75; 87)	100	0	0	0
	CLO	6	1975 ± 1810	2092 ± 943	1 (0; 13)	54	46	0	0
	MOX	7	1750 ± 1632	1314 ± 1818	24 (10; 37)	57	42	1	0
8	CON	9	6356 ± 6540	5778 ± 7481	–	81	5	11	3
	ALB	9	6711 ± 6671	2938 ± 3479	50 (43; 54)	73	19	8	0
	IVM	9	7050 ± 7567	3956 ± 3025	44 (38; 48)	90	10	0	0
	LEV	9	6461 ± 6925	2578 ± 2787	60 (56; 64)	88	12	0	0
	MPT	9	7622 ± 10,151	344 ± 366	95 (94; 97)	88	0	0	12
	CLO	8	8013 ± 11,040	844 ± 1004	89 (87; 91)	8	27	64	1
	MOX	8	6850 ± 8565	7175 ± 9569	0 (0; 5)	78	22	0	0
9	CON	8	1538 ± 897	2800 ± 3831	–	71	29	0	0
	ALB	8	1719 ± 1759	944 ± 842	44 (33; 55)	83	16	1	0
	IVM	8	1700 ± 1448	381 ± 287	78 (70; 83)	88	11	0	0
	LEV	8	1756 ± 1503	656 ± 577	63 (53; 70)	66	34	0	0
	MPT	8	1656 ± 1322	113 ± 171	93 (89; 96)	2	0	0	98
	CLO	8	1750 ± 1115	500 ± 255	72 (64; 78)	16	67	3	14
	MOX	8	1625 ± 1091	1388 ± 1183	13 (1; 27)	48	52	0	0
10	CON	8	2413 ± 2488	4519 ± 3540	–	9	88	0	3
	ALB	8	2025 ± 2257	3163 ± 3434	0 (0; 3)	7	93	0	0
	IVM	8	2150 ± 2249	4971 ± 2613	0 (0; 1)	2	98	0	0
	LEV	8	2138 ± 2293	1000 ± 2259	54 (43; 61)	12	88	0	0
	MPT	8	2150 ± 2175	469 ± 688	78 (72; 83)	90	0	0	10
	CLO		*	*	*	*	*	*	*
	MOX		*	*	*	*	*	*	*
11	CON	8	1819 ± 1523	1244 ± 1210	–	99	1	0	0
	ALB	8	1788 ± 1848	1044 ± 936	41 (29; 52)	91	9	0	0
	IVM	8	1775 ± 1606	1681 ± 2117	6 (0; 18)	94	6	0	0
	LEV	8	1775 ± 1842	706 ± 892	60 (51; 68)	95	5	0	0
	MPT	8	1719 ± 1509	6 ± 18	99 (98; 100)	0	0	0	0
	CLO	8	1700 ± 1439	319 ± 340	81 (75; 86)	39	61	0	0
	MOX	8	1700 ± 1389	1650 ± 1150	3 (0; 17)	91	9	0	0
12	CON	8	1156 ± 1025	1093 ± 465	–	34	13	53	0
	ALB	8	1238 ± 1302	514 ± 400	62 (51; 72)	46	15	39	0
	IVM	8	1225 ± 1143	250 ± 431	74 (64; 83)	90	10	0	0
	LEV	8	1206 ± 1275	100 ± 171	89 (81; 94)	73	9	18	0
	MPT	8	1200 ± 1065	263 ± 320	78 (70; 84)	44	0	1	55
	CLO		*	*	*	*	*	*	*
	MOX		*	*	*	*	*	*	*
13	CON	8	969 ± 703	888 ± 640	–	71	29	0	0
	ALB	8	1131 ± 1265	263 ± 169	77 (68; 84)	76	24	0	0
	IVM	8	1013 ± 930	788 ± 650	22 (3; 37)	95	5	0	0
	LEV	8	994 ± 956	1213 ± 1777	1 (0; 11)	92	8	0	0
	MPT	8	1025 ± 881	56 ± 68	95 (90; 97)	100	0	0	0
	CLO		*	*	*	*	*	*	*
	MOX		*	*	*	*	*	*	*
14	CON	8	1888 ± 1603	1594 ± 1240	–	68	17	0	15
	ALB	8	1706 ± 1259	1056 ± 582	38 (25; 49)	98	2	0	0
	IVM	8	1831 ± 1880	1681 ± 1973	8 (0; 2)	99	0	0	1
	LEV	8	1800 ± 2623	556 ± 457	69 (61; 76)	89	11	0	0
	MPT	8	1844 ± 1854	456 ± 323	75 (68; 81)	88	0	0	12
	CLO		*	*	*	*	*	*	*
	MOX		*	*	*	*	*	*	*
15	CON	7	5250 ± 4270	1643 ± 1813	–	99	1	0	0
	ALB	7	5336 ± 5569	1721 ± 2198	68 (63; 72)	100	0	0	0
	IVM	7	4957 ± 5621	2571 ± 3861	47 (41; 54)	99	1	0	0
	LEV	7	5564 ± 5815	1536 ± 2912	72 (68; 76)	100	0	0	0
	MPT	7	5221 ± 4702	36 ± 38	99 (99; 100)	100	0	0	0
	CLO		*	*	*	*	*	*	*
	MOX		*	*	*	*	*	*	*

*n*: number of animals; H: *Haemonchus* spp.; T: *Trichostrongylus* spp.; C: *Cooperia* spp.; O: *Oesophagostomum* spp.; CON: control; ALB: albendazole; IVM: ivermectin; LEV: levamisole; MPT: monepantel; CLO: closantel; MOX: moxidectin; -: not evaluated and *: insufficient number of animals for these groups.

All farms showed albendazole resistance, with efficacy varying from 0 in three farms (Farms 3, 4, and 10) to a maximum of 79% (Farm 5) with an average of 40% in the FECR among all farms (Table 1 and Fig. 2). Based on faecal cultures, *Haemonchus* spp. was the major albendazole resistant nematode with 10% of average reduction, followed, to a lesser extent, by *Trichostrongylus* spp. (38% of reduction). Exceptions were detected in Farms 2 and 7, presenting a higher frequency of *Trichostrongylus* after albendazole treatment, and in Farm 2, with a sharp increase in *Cooperia* spp. L_3_ (Table 1, Table 2).

**Table 2:** Mean percentage of efficacy against each nematode genus ±standard deviation after anthelmintic treatment.

Anthelmintics	% efficacy against each nematode genus			
	H	T	C	O
ALB	10 ± 22.5	38 ± 42.1	45 ± 36.5	99 ± 3.4
IVM	14 ± 29.8	24 ± 36.0	80 ± 40.0	99 ± 2.6
LEV	9 ± 21.8	22 ± 31.0	90 ± 15.5	100 ± 0.0
MPT	25 ± 39.7	100 ± 0.0	62 ± 49.1	*
CLO	45 ± 44.2	*	*	*
MOX	8 ± 11.1	13 ± 35.5	33 ± 57.7	100 ± 0.0

ALB: albendazole; IVM: ivermectin; LEV: levamisole; MPT: monepantel; CLO: closantel; MOX: moxidectin; H: *Haemonchus* spp.; T: *Trichostrongylus* spp.; C: *Cooperia* spp.; O: *Oesophagostomum* spp.; *: there is no claim of MPT efficacy against O and CLO against T, C and O.

In general, levamisole also presented low efficacy rates (48% on average, Fig. 2). The best results in efficacy were observed in Farms 1 (90%) and 12 (89%), whereas on the other 13 farms, efficacy varied from 0 to 79% (Table 1). *Haemonchus* spp. L_3_ was the most prevalent resistant nematode in most farms with a mean reduction of 9% (Table 2), except for Farms 2, 4, and 10, where *Trichostrongylus* spp. L_3_ prevailed (82%, 52%, and 88%, respectively, Table 1).

Ivermectin demonstrated a mean efficacy of 34% across farms (Table 1, Fig. 2). Notably, four farms recorded 0% efficacy. The predominant L_3_ genera identified in faecal cultures were *Haemonchus* spp., except for Farms 2 and 10, where 95% and 98%, respectively, of *Trichostrongylus* spp. were detected.

Similarly to ivermectin, moxidectin had the worst general efficacy mean (21%) among the 7 farms where it was evaluated (Table 1, Fig. 2). *Haemonchus* spp. L_3_ was the most prevalent genus in most of the farms, except for Farm 9, where *Trichostrongylus* spp. prevailed (52%, Table 1, Fig. 2). The worst efficacies were observed against moxidectin with the lowest reductions in the L_3_ percentage of *Haemonchus* spp., *Trichostrongylus* spp. and *Cooperia* spp. (Table 2).

Presenting the best efficacies, monepantel showed a 79% mean FECR, but only in Farms 6, 8, 11, 13, and 15 its efficacy was ≥95% (Fig. 2). Most of the larvae produced in faecal cultures post-treatment with monepantel were *Haemonchus* spp. and *Oesophagostomum* spp., with a few *Cooperia* spp. (Table 1, Table 2). It calls attention to the relatively high proportions (>50%) of *Oesophagostomum* larvae in farms 2, 3, 6, 9 and 12 (Table 1). The efficacy of monepantel against *Trichostrongylus* spp. was 100% (Table 2).

Anthelmintic resistance to closantel was observed in all nine farms where the compound was assessed, with a mean efficacy of 45% against *Haemonchus* spp. and 63% on the FECRT (Table 1, Table 2 and Fig. 2).

FECRT revealed that all 15 sheep flocks presented gastrointestinal nematodes with multiple anthelmintic resistance (Table 1, Table 2 and Fig. 2).

Comparing the present study with previous surveys developed in Sao Paulo state it is possible to note that situation has worsened: in the first survey, *Trichostrongylus* spp. showed resistance only to levamisole and the most recent studies also to benzimidazole, ivermectin and moxidectin; *Cooperia* spp. has become resistant to albendazole and moxidectin and less anthelmintics have achieved efficacy >90% (Table 3).

**Table 3:** Reports of anthelmintic resistance based on faecal egg count reduction tests in sheep of São Paulo state.

Anthelmintics	Year	Farms	FECR			Main resistant nematodes	Source
			<50%	50%–90%	>90%		
BNZ	1990	9	9	0	0	H	Amarante et al. (1992)
	2008–2010	30	25	5	0	H; T	Veríssimo et al. (2012)
	2018–2020	5	4	1	0	–	Gainza et al. (2021)
	2022	15	9	6	0	H; T; C	Present study

IVM	1990	9	1	6	2	H	Amarante et al. (1992)
	2008–2010	28	20	8	0	H; T	Veríssimo et al. (2012)
	2018–2020	5	5	0	0	–	Gainza et al. (2021)
	2022	15	10	5	0	H; T	Present study

LEV	1990	9	1	6	2	H; T	Amarante et al. (1992)
	2008–2010	28	9	6	13	H; T	Veríssimo et al. (2012)
	2018–2020	5	2	3	0	–	Gainza et al. (2021)
	2022	15	5	10	0	H; T	Present study

MPT	2018–2020	5	0	2	3	–	Gainza et al. (2021)
	2022	15	2	7	6	H	Present study

CLO	2008–2010	28	13	13	2	H	Veríssimo et al. (2012)
	2022	9	2	6	1	H	Present study

MOX	2008–2010	28	21	6	1	H; T	Veríssimo et al. (2012)
	2022	7	6	1	0	H; T; C	Present study

FECR: Faecal egg count reduction post-treatment; BNZ: benzimidazole; IVM: ivermectin; LEV: levamisole; MPT: monepantel; CLO: closantel; MOX: moxidectin; -: data not presented; H: *Haemonchus* spp.; T: *Trichostrongylus* spp.; C: *Cooperia* spp.

## 4 Discussion

Over the years, farmers have changed the management practices from drenching every 30 days, as usual in the 90's, to targeted selective treatment or drenching the animals when presenting bottle jaw. Unfortunately, even using lesser drenches, the situation has worsened in comparison with previous surveys performed in São Paulo state as demonstrated in Table 3 (Amarante et al., 1992; Veríssimo et al., 2012). Even with the use of targeted selective treatment, there have been reports of the rapid emergence of monepantel-resistant *Haemonchus* populations (Mederos et al., 2014; Albuquerque et al., 2017). Chagas et al. (2016) reported that in a sheep flock, after five years of exclusive use of levamisole in a targeted selective treatment scheme, the initial efficacy, which was 100%, was reduced to 65.5%, based on FECRT. The maintenance of animals susceptible to parasites in the flocks, receiving frequent treatments, is one of the plausible explanations for the targeted selective treatment failure in preventing the emergence of resistant nematode populations.

In the farms presenting monepantel susceptibility (as in Farm 11), the farmers informed that this anthelmintic has not been used for a long time or has never been used. Monepantel (Zolvix®) has been sold in Brazil from 2012 to 2021, showing high efficacy in evaluations carried out on five farms in São Paulo State (Gainza et al., 2021). However, several cases of resistance to monepantel in *Haemonchus* have been reported in Brazil (Albuquerque et al., 2017; Martins et al., 2017; Oliveira et al., 2017; Ramos et al., 2018; Silva et al., 2018; Gainza et al., 2021). *Haemonchus* populations showed rapid development of resistance (Mederos et al., 2014), and, in all farms evaluated here, it was possible to detect a rise in *Haemonchus* resistance to monepantel. Frequent movement of animals without carrying out adequate quarantine before introducing sheep into the herd might also favour the transfer of resistant parasites among farms. This is clear considering the results of Farm 15 that started to raise sheep only six months before the present study: Of the four anthelmintics tested, only monepantel was effective. In this case, certainly the animals were acquired already infected with resistant parasites from the farm of origin. Regarding monepantel results, apart from two properties, monepantel was the anthelmintic that showed greater effectiveness. We ascribed its higher efficacy to limited use, mainly because monepantel was so costly for the conditions of Brazilians’ farmers that it was not widely utilised in the country.

Amarante et al. (1992) evaluated anthelmintic resistance against oxfendazole, levamisole, and ivermectin in nine sheep flocks in Sao Paulo State in 1990. In most farms, multiple anthelmintic resistance was reported, but two farms showed FECR superior to 90% after levamisole treatment and two after ivermectin treatment. At that moment, deworming was usually done every 30 days. *Haemonchus* spp. (78–100%) was the major genera in L_3_ from faecal cultures, followed by *Trichostrongylus* spp. (0–16%). Oxfendazole (used by Amarante et al., 1992) and albendazole (used in the present trial) are from the same group of benzimidazoles, and it is possible to see that they have been ineffective in reducing FEC for more than 30 years. In Farm 2, according to the farmer, this product has not been used for more than 5 years, and even though its efficacy is still low (52%). Taken together, these observations indicate that effective resistance reversal is not observed even after a long time without the use of benzimidazoles.

In another survey carried out from 2008 to 2010 in Sao Paulo State, albendazole and ivermectin were ineffective on all 30 farms assessed. Gastrointestinal nematodes displayed resistance to levamisole in 53.6% of the farms, to closantel in 92.2%, and to moxidectin in 96.6% (Veríssimo et al., 2012). Comparatively, in the current study, the efficacy of all anthelmintics became worse, with levamisole and closantel showing no efficacy currently.

The most recent research done in Sao Paulo State evaluating five farms reported that monepantel was the only effective anthelmintic, probably due to its low use in the country. Albendazole and ivermectin showed the worst results, and levamisole presented efficacy of 81% and 89% in two farms (Gainza et al., 2021). These results corroborate with ours with poor anthelmintic efficacy, meanwhile levamisole presenting the best FECR.

In Farm 8, a FECRT conducted in 2016 (unpublished data) obtained an efficacy of 59% for albendazole + levamisole (10 mg/kg BW + 9.4 mg/kg BW), 98% for monepantel (2.5 mg/kg BW), 76% for closantel (10 mg/kg BW), and 40% for moxidectin (0.4 mg/kg BW). It is important to mention that the doses of albendazole, levamisole, and moxidectin used in 2016 were higher than the recommended doses, and this may explain the higher efficacies recorded in 2016.

There are two reports of *Trichostrongylus* resistance to monepantel (Scott et al., 2013; Cintra et al., 2016), in which the resistance status was confirmed after worm burden counting post euthanasia. While a high prevalence (94%) of *Trichostrongylus* L_3_ was detected by Scott et al. (2013), no *Trichostrongylus* L_3_ was found in faecal cultures by Cintra et al. (2016). This indicates a limitation of the FECRT to detect *Trichostrongylus* resistance in mixed-infected sheep, which may show a predominance of *Haemonchus* larvae in faecal cultures. The production of eggs from a *Haemonchus* female (around 5000 eggs/day) is much higher than the production of eggs from *Trichostrongylus* (200 eggs/day). Consequently, *Haemonchus* larvae will be the most prevalent in faecal cultures of mixed-infected sheep. Almeida et al. (2010), also did not find any L_3_ of *T. colubriformis* in faecal culture, however, based on the results of the controlled efficacy test, they reported the first case of resistance of this parasite to macrocyclic lactones in Brazil. For this reason, results considering *T. colubriformis* susceptibility should be evaluated with caution. Therefore, in the present survey, we cannot discard the possibility of detecting *Trichostrongylus* resistance at a higher frequency, including monepantel, if worm counting were performed. “Nemabiome”, which allows the identification and quantification of gastrointestinal nematode species in a worm population (Avramenko et al., 2017), could be an option to avoid the misinterpretation of this sort of result, but this technology is still costly and not available in all countries.

In Brazil, most farmers do not have accurate information about the resistance status of the anthelmintics used on their properties. The antiparasitic choice generally depends on price, advertising, and sellers' recommendations. These factors explain why farmers continue to use ivermectin and moxidectin, when parasitic resistance to these drugs was already detected in the 90s. A recently published work about farmers' economic decisions in Sweden on testing and treatment of livestock diseases indicated that it is more profitable to treat animals without prior FECRT. This approach can be a problem as it could result in overuse of anthelmintics, potentially worsening the anthelmintic resistance, resulting in a negative impact on sheep production (Aklilu et al., 2024).

The indiscriminate use of anthelmintics without monitoring their efficacy through regular parasitological exams has proven to be ineffective. It is imperative to shift focus towards enhancing management practices that promote better nutrition and reduce animals' exposure to severe infections. Additionally, selective breeding for sheep with a higher capacity to resist infections should be prioritized, with particular attention given to local breeds that exhibit natural resistance to haemonchosis.

## 5 Conclusion

In conclusion, it is evident that the status of anthelmintic resistance in sheep flocks in the State of São Paulo has not shown any improvement over the last 30 years. Instead, the situation has worsened, with evidence pointing to a higher and more widespread resistance of nematodes against multiple anthelmintic molecules. Due to the limited options of anthelmintics available and widespread resistance, this situation shows that sheep farmers need to put into practice innovative and sustainable strategies to control gastrointestinal nematode infection.

## Financial support statement

**Cesar C. Bassetto** received financial support from 10.13039/501100001807FAPESP (grant #2020/13972-4 and grant #2024/02124-3, São Paulo Research Foundation).

**Ana Cláudia A. Albuquerque** received financial support from 10.13039/501100001807FAPESP (grant #2021/03479-1, São Paulo Research Foundation).

**Jose Gabriel G. Lins** received financial support from 10.13039/501100002322CAPES (10.13039/501100002322Coordination for the Improvement of Higher Education Personnel).

**Naiara M. Marinho-Silva** received financial support from 10.13039/501100002322CAPES.

**Marianna L. E. Chocob**ar received financial support from 10.13039/501100003593CNPq (10.13039/501100003593National Council for Scientific and Technological Development, #161949/2021-5).

**Hornblenda J. S. Bello** received financial support from 10.13039/501100002322CAPES.

**Mateus O. Mena** received financial support from 10.13039/501100002322CAPES.

**Alessandro F. T. Amarante** received financial support from 10.13039/501100003593CNPq (#303624/2021-3).

**Ana Carolina S. Chagas** received financial support from 10.13039/501100001807FAPESP (grant #2019/02929-3, São Paulo Research Foundation).

## Data and model availability statement

None of the data were deposited in an official repository. However, the data that support the study findings are available from authors upon request.

## Declaration of generative AI in scientific writing

The authors did not use any artificial intelligence-assisted technologies in the writing process.

## Author contributions

**C. C. Bassetto:** conceptualization, formal analysis, investigation, data curation, writing - original draft, writing - review & editing, project administration, funding acquisition. **A. C. A. Albuquerque** and **J. G. G. Lins:** investigation, writing - original draft, writing - review & editing. **N. M. Marinho-Silva, M. L. E. Chocobar, H. J. S. Bello** and **M. O. Mena:** investigation, writing - review & editing. **S. C. M. Niciura**: writing - original draft, writing - review & editing. **A. F. T. Amarante**: conceptualization, formal analysis, resources, writing - original draft, writing - review & editing, supervision. **A. C. S. Chagas**: conceptualization, resources, data curation, writing - original draft, writing - review & editing, supervision, project administration, funding acquisition.

## Declaration of competing interest

None.

## References

[bib1] Aklilu A.Z., Elofsson K., Halvarsson P., Kjellander P., Höglund J. (2024). A pound for information or a pence for cure: farmers' economic decisions on testing and treatment of livestock diseases. Aust. J. Agric. Resour. Econ..

[bib2] Albuquerque A.C.A., Bassetto C.C., Almeida F.A., Amarante A.F.T. (2017). Development of *Haemonchus contortus* resistance in sheep under suppressive or targeted selective treatment with monepantel. Vet. Parasitol..

[bib3] Almeida F.A., Garcia K.C.O.D., Torgerson P.R., Amarante A.F.T. (2010). Multiple resistance to anthelmintics by *Haemonchus contortus* and *Trichostrongylus colubriformis* in sheep in Brazil. Parasitol. Int..

[bib4] Almeida F.A., Bassetto C.C., Amarante M.R.V., Albuquerque A.C.A., Starling R.Z.C., Amarante A.F.T. (2018). Helminth infections and hybridization between *Haemonchus contortus* and *Haemonchus placei* in sheep from Santana do Livramento, Brazil. Rev. Bras. Parasitol. Vet..

[bib5] Almeida F.A., Albuquerque A.C.A., Bassetto C.C., Starling R.Z.C., Lins J.G.G., Amarante A.F.T. (2020). Long spelling periods are required for pasture to become free of contamination by infective larvae of *Haemonchus contortus* in a humid subtropical climate of São Paulo state, Brazil. Vet. Parasitol..

[bib6] Amarante A.F.T., Barbosa M.A., Oliveira M.A.G., Carmello M.J., Padovani C.R. (1992). Efeito da administração de oxfendazol, ivermectina e levamisol sobre os exames coproparasitológicos de ovinos. Braz. J. Vet. Res. Anim. Sci..

[bib7] Amarante A.F.T., Bricarello P.A., Rocha R.A., Gennari S.M. (2004). Resistance of Santa Ines, Suffolk, and Ile de France sheep to naturally acquired gastrointestinal nematode infections. Vet. Parasitol..

[bib8] Avramenko R.W., Redman E.M., Lewis R., Bichuette M.A., Palmeira B.M., Yazwinski T.A., Gilleard J.S. (2017). The use of nemabiome metabarcoding to explore gastro-intestinal nematode species diversity and anthelmintic treatment effectiveness in beef calves. Int. J. Parasitol..

[bib9] Besier R.B., Kahn L.P., Sargison N.D., Van Wyk J.A. (2016). Chapter four - the pathophysiology, ecology and epidemiology of *Haemonchus contortus* infection in small ruminants. Adv. Parasitol..

[bib10] Carvalho N., Neves J.H., Pennacchi C.S., Castilhos A.M., Amarante A.F.T. (2021). Performance of lambs under four levels of dietary supplementation and artificially mix-infected with *Haemonchus contortus* and *Trichostrongylus colubriformis*. Rev. Bras. Parasitol. Vet..

[bib11] Chagas A.C.S., Domingues L.F., Gaínza Y.A., Barioni-Júnior W., Esteves S.N., Niciura S.C.M. (2016). Target selected treatment with levamisole to control the development of anthelmintic resistance in a sheep flock. Parasitol. Res..

[bib12] Cintra M.C.R., Teixeira V.N., Nascimento L.V., Sotomaior C.S. (2016). Lack of efficacy of monepantel against *Trichostrongylus colubriformis* in sheep in Brazil. Vet. Parasitol..

[bib13] Cristel S., Fiel C., Anziani O., Descarga C., Cetrá B., Romero J., Fernández S., Entrocasso C., Lloberas M., Medus D., Steffan P. (2017). Anthelmintic resistance in grazing beef cattle in central and northeastern areas of Argentina — an update. Vet. Parasitol. Reg. Stud. Reports..

[bib14] Coles G.C., Jackson F., Pomroy W.E., Prichard R.K., von Samson-Himmelstjerna G., Silvestre A., Taylor M.A., Vercruysse J. (2006). The detection of anthelmintic resistance in nematodes of veterinary importance. Vet. Parasitol..

[bib15] Gainza Y.A., Santos I.B., Figueiredo A., Santos L.A.L., Esteves S.N., Barioni-Junior W., Minho A.P., Chagas A.C.S. (2021). Anthelmintic resistance of *Haemonchus contortus* from sheep flocks in Brazil: concordance of in vivo and in vitro (RESISTA-Test©) methods. Rev. Bras. Parasitol. Vet..

[bib16] Gilleard J.S., Kotze A.C., Leathwick D., Nisbet A.J., McNeilly T.N., Besier B. (2021). A journey through 50 years of research relevant to the control of gastrointestinal nematodes in ruminant livestock and thoughts on future directions. Int. J. Parasitol..

[bib17] Kaplan R.M., Denwood M.J., Nielsen M.K., Thamsborg S.M., Torgerson P.R., Gilleard J.S., Dobson R.J., Vercruysse J., Levecke B. (2023). World Association for the Advancement of Veterinary Parasitology (W.A.A.V.P.) guideline for diagnosing anthelmintic resistance using the faecal egg count reduction test in ruminants, horses, and swine. Vet. Parasitol..

[bib18] Kimambo A.E., MacRae J.C., Dewey P.J. (1988). The effect of daily challenge with *Trichostrongylus colubriformis* larvae on the nutrition and performance of immunologically resistant sheep. Vet. Parasitol..

[bib19] Leathwick D.M., Ganesh S., Waghorn T.S. (2015). Evidence for reversion towards anthelmintic susceptibility in *Teladorsagia circumcincta* in response to resistance management programmes. Int. J. Parasitol. Drugs Drug Resist..

[bib20] Macedo L.O., Silva S.S., Alves L.C., Carvalho G.A., Ramos R.A.N. (2023). An overview of anthelmintic resistance in domestic ruminants in Brazil. Ruminants.

[bib21] Martins A.C., Bergamasco P.L.F., Felippelli G., Tebaldi J.H., Moraes M.F.D., Testi A.J.P., Lapera I.M., Hoppe E.G.L. (2017). *Haemonchus contortus* resistance to monepantel in sheep: fecal egg count reduction tests and randomized controlled trials. Semina Ciências Agrárias.

[bib22] Mederos A.E., Ramos Z., Banchero G.E. (2014). First report of monepantel *Haemonchus contortus* resistance on sheep farms in Uruguay. Parasites Vectors.

[bib23] Nagata W.B., Panegossi M.F.C., Bresciani K.D.S., Gomes J.F., Kaneto C.N., Perri S.H.V. (2019). Resistance of gastrointestinal nematodes to five different active principles in sheep infected naturally in São Paulo State, Brazil. Small Rumin. Res..

[bib24] Neves J.H., Carvalho N., Rinaldi L., Cringoli Giuseppe, Amarante A.F.T. (2014). Diagnosis of anthelmintic resistance in cattle in Brazil: a comparison of different methodologies. Vet. Parasitol..

[bib25] Oliveira P.A., Riet-Correa B., Estima-Silva P., Coelho A.C.B., Santos B.L., Costa M.A.P., Ruas J.L., Schild A.L. (2017). Multiple anthelmintic resistance in Southern Brazil sheep flocks. Rev. Bras. Parasitol. Vet..

[bib26] Ramos F., Portella L.P., Rodrigues F.S., Reginato C.Z., Cezar S.A., Sangioni L.A., Vogel F.S.F. (2018). Anthelminthic resistance of gastrointestinal nematodes in sheep to monepantel treatment in central region of Rio Grande do Sul, Brazil. Pesqui. Vet. Bras..

[bib27] Rocha R.A., Amarante A.F.T., Bricarello P.A. (2004). Comparison of the susceptibility of Santa Inês and Ile de France ewes to nematode parasitism around parturition and during lactation. Small Rumin. Res..

[bib28] Salgado J.A., Santos C.P. (2016). Overview of anthelmintic resistance of gastrointestinal nematodes of small ruminants in Brazil. Rev. Bras. Parasitol. Vet..

[bib29] Santos M.C., Xavier J.K., Amarante M.R.V., Bassetto C.C., Amarante A.F.T. (2014). Immune response to *Haemonchus contortus* and its role on parasite specificity. Vet. Parasitol..

[bib30] Santos I.B., Anholeto L.A., Sousa G.A., Nucci A.S., Gainza Y.A., Figueiredo A., Santos L.A.L., Minho A.P., Barioni-Junior W., Esteves S.N., Niciura S.C.M., Chagas A.C.S. (2022). Investigating the benefits of targeted selective treatment according to average daily weight gain against gastrointestinal nematodes in Morada Nova lambs. Parasitol. Res..

[bib31] Scott I., Pomroy W.E., Kenyon P.R., Smith G., Adlington B., Moss A. (2013). Lack of efficacy of monepantel against *Teladorsagia circumcincta* and *Trichostrongylus colubriformis*. Vet. Parasitol..

[bib32] Silva F.F., Bezerra H.M.F.F., Feitosa T.F., Vilela V.L.R. (2018). Nematode resistance to five anthelmintic classes in naturally infected sheep herds in Northeastern Brazil. Rev. Bras. Parasitol. Vet..

[bib33] Torgerson P.R., Paul M., Furrer R. (2014). Evaluating faecal egg count reduction using a specifically designed package ‘‘eggCounts’’ in R and a user-friendly web interface. Int. J. Parasitol..

[bib34] Ueno H., Gonçalves P.C. (1998).

[bib35] van Wyk J.A., Bath G.F. (2002). The FAMACHA© system for managing haemonchosis in sheep and goats by clinically identifying individual animals for treatment. Vet. Res..

[bib36] Veríssimo C.J., Niciura S.C.M., Alberti A.L.L., Rodrigues C.F.C., Barbosa C.M.P., Chiebao D.P., Cardoso D., Silva G.S., Pereira J.R., Margatho L.F.F., Costa R.L.D., Nardon R.F., Ueno T.E.H., Curci V.C.L.M., Molento M.B. (2012). Multidrug and multispecies resistance in sheep flocks from São Paulo state, Brazil. Vet. Parasitol..

[bib37] Waller P.J. (1997). Anthelmintic resistance. Vet. Parasitol..

[bib38] Wilmsen M.O., Silva B.F., Bassetto C.C., Amarante A.F.T. (2014). Gastrointestinal nematode infections in sheep raised in Botucatu, state of São Paulo, Brazil. Rev. Bras. Parasitol. Vet..

